# Reliability and validity of the Arabic version of the biological rhythms interview of assessment in neuropsychiatry

**DOI:** 10.1002/npr2.12273

**Published:** 2022-06-13

**Authors:** Odeh S. Murad, Khaled A. Al‐Dassean

**Affiliations:** ^1^ Al‐Balqa Applied University, Al‐Shoubak University College Al Shoubak Jordan

**Keywords:** biological rhythms, BRIAN, circardian, depression, sleep disorders

## Abstract

**Background:**

Disruptions in biological rhythm (BR) are considered a factor in the spread of many chronic diseases such as hypertension, diabetes, and depression. It has been shown that imbalance in BR disrupts the body’s physiological timings; therefore, it is essential to have a tool for BR evaluation.

**Methods:**

A cross‐sectional study was conducted on a sample of 403 Jordanian participants (200 depressed people and 203 control groups). Classical test theory (CTT) was used to evaluate the psychometric properties of the Arabic version of BRIAN. We aimed to validate the Arabic version of Biological Rhythms Interview Assessment in Neuropsychiatry (BRIAN) by investigating its internal consistency and validity, assessing its factor structure, and exploring its relationships with depression and sleep disorders.

**Results:**

The internal consistency (*α*) was 0.91. The concurrent validity was supported by the severity of depression and sleep disorders (*r* = 0.87, *r* = 0.83, *p* < 0.001). The BRIAN’s ability to differentiate between depressed people and the control group supported its discriminant validity (*t* = 21.2, *p* = 0.001). With a sensitivity of 75 and a specificity of 95.57, BRIAN revealed good accuracy in distinguishing between depressed and non‐depressed persons at cutoff 44. The exploratory factor analysis (EFA) and the confirmatory factor analysis (CFA) analyses supported its proposed three‐factor solutions.

**Conclusions:**

The results demonstrated that the BRIAN‐A has acceptable validity in detecting BR and could be useful in examining the impact of circadian disturbance on the Arabic population.

**Abbreviations**
BRIAN: Biological Rhythms Interview Assessment in Neuropsychiatry
AUC: Area Under the Curve

## 1 INTRODUCTION

The coronavirus pandemic has forced the world to undergo many unavoidable challenges and has prompted it to live with this epidemic according to circumstances and data different from what the world has known before. The pandemic forced governments in various countries of the world to race against time to take the necessary steps to limit the spread of COVID‐19.^1^ Among these plans include the disruption of and lockdown of educational institutions such as schools, universities, and all sectors, except for the essential and health sectors, in line with the health protocol launched by the World Health Organization, which had called for partial or comprehensive prohibitions, the maintenance of a safe distance from others to ensure social distancing, wearing of masks and gloves, and making sure to use sterilizers and disinfectants.^2^

In this pandemic period of home isolation, lockdown, and sleep disorders for many people, boredom increased, and most people resorted to staying up late watching TV and movies.^3^ When staying up late becomes a daily habit, lifestyle, work productivity, mental and physical health could be affected,^4^ which is classified as a psychiatric diagnosis called biological rhythms disorder. It is necessary to maintain the quality of sleep at night to ensure control of biological rhythms.^5^

Mental health experts have explained that the melatonin hormone is responsible for the biological rhythm, as it is secreted by the gland and brain from 9 pm to 3 am and works during the evening period.^6^ Therefore, a person must sleep during that period to benefit from the hormone. Thus, adjusting adequate sleep hours, entering deep sleep, and not disturbing the sleep helps a lot in human health, improving the immune and nervous systems.^4^

To maintain the quality of sleep, one should avoid sleeping for long hours during the day, not drink stimulants after sunset, and stay away from devices with screen lights, such as mobile and television, before bedtime, to avoid disturbing the biological clock.^7^

Circadian rhythm is defined as “the period of physiological function and behavior of the organism in about 24 hours, and this rhythm includes various physiological functions such as sleep‐wake cycle, body temperature, regulation and secretion of hormones, cell proliferation, and digestive function.”.^8^ Psychological and social rhythm can influence the circadian rhythm.^6^

The literature indicated an association between circadian rhythm disturbances, psychological disorders, poor performance, and quality of life.^9^, ^10^ The literature also indicates that circadian disturbances are greater in people with higher depression compared to people with less depression.^11^

Depression is a recurrent and chronic disorder. Wilson et al^12^ with a broad range of manifestations of psychological distress and functional deficits to deteriorating physical health. Depression is one of the leading causes of disability in the world, and it is expected to be the leading contributor to the global disease burden.^13^, ^14^, ^15^ Certain areas of biological rhythms, such as sleep, activity, social rhythm, and eating patterns may have a significant impact on the severity of depression. As a result, they should be considered in depression treatment.^16^

The most important distinguishing features of major depression and disorders are abnormal sleep–wake cycles, lack of appetite, hunger, lack of energy, ability to focus, and mood itself.^3^ These rhythms are disturbed by external factors that may lead to mood attacks in those individuals.^17^ As a result of the external factors that the COVID‐19 pandemic added, such as the unprecedented habits that individuals were forced to take up, for example, home quarantine, social distancing because of closures, wearing masks, and washing hands, there arose disturbances in circadian rhythms, and the severity of depression and psychological disorders increased.^18^

Giglio et al^19^ developed the Biological Rhythms Interview of Assessment in Neuropsychiatry (BRIAN) to investigate the severity of disturbances in the areas of the biological rhythm. The BRIAN is a tool for assessing circadian rhythms,^19^ and it has been used in a number of studies. Moro et al^10^ found that patients with bipolar disorder scored significantly higher on this scale than the control group when using the Italian version of BRIAN. In a Spanish study, patients with remitting bipolar disorder were found to have more alterations in biological systems than the control group and to be more impaired in the domains of sleep and activity.^20^ Patients with bipolar disorder and major depression had more circadian rhythm disturbance than the control group in a Brazilian study.^21^ A more recent study found that the Polish version of the BRIAN scale had high feasibility and consistency, indicating that patients had more biological rhythm disturbances than controls. Higher BRIAN scores have a positive correlation with morningness and eveningness, but the correlations with vigilance and ability to stay awake are negative.^33^

Previous studies that used BRIAN found that biological rhythm disturbances affect psychosocial and social functioning, the severity of depression, and the quality of life.^9^, ^22^, ^23^ Given the paucity of local studies on this topic and the lack of a psychological instrument to measure biological circadian rhythms. To the best of the researchers' knowledge, no Arabic research on the BRIAN has been found. So the current study was carried out by the researchers in order to validate the Arabic version of BRIAN and investigate its psychometric properties.

### 1.1 Aim of the study

The current study aimed to validate the Arabic version of the BRIAN for the people who had to undertake home quarantine by (1) investigating its internal consistency, (2) examining its proposed factor structure, and (3) e its relationships with depression and sleep disorders.

### 1.2 Research hypothesis

Hypothesis 1: the Arabic version of BRIAN will show a good (>0.8) internal consistency reliability as measured by Cronbach’s alpha.

Hypothesis 2: the Arabic version of BRIAN will show the same proposed factor structure as in the original version.

Hypothesis 3: the Arabic version of BRIAN would positively correlate with Depression and Sleep Disorders.

## 2 METHODS

### 2.1 Participants

The present study focused on people who had to undertake home quarantine for 14–28 days, such as university students whose universities had shut down and people who had lost their jobs because of the lockdown to keep COVID‐2019 from spreading. The sample consisted of 403 individuals (200 depressed, 203 in control groups). Using the psychometric method in the diagnosis of depression, we defined and selected the depressed group according to their scores on the Beck Depression Inventory (BDI‐II). The cut‐off point 39 (of 63) was arbitrarily chosen to select the participants for this group.^24^ This was done considering the exceptional circumstances of the participants because of the lockdown and to distinguish them from the control group that also lived in the same conditions. The control group participants were selected based on their low scores on the BDI‐II. All of them reported that they were not depressed and had not suffered from any depressive symptoms in the past 14 days. Participants who did not match the criteria were excluded. The data were collected from May 2, 2020 to May 22, 2020.

The consent form, which the participants approved, ensured complete confidentiality of their identities and data. They were also given information about the study’s purpose and procedures. They were asked not to write their names and had the right to withdraw their data at any time they wanted. Gender, age, educational level, marital status, and employment position were all included in the demographic data. Online advertisements, social media, and online blogs were used to attract participants. A link was sent using Google Forms to 500 people, 403 responded, all of whom met the required criteria and no one was excluded.

### 2.2 Measures

#### 2.2.1 Biological Rhythms Interview of Assessment in Neuropsychiatry (BRIAN)

BRIAN is an interview developed by Giglio et al^19^ which includes 18 items that assess sleep, activities, social rhythms, and eating habits 18 items that evaluate sleep, activities, social rhythm, and eating patterns. The items are rated on a four‐point scale from 1 = not at all to 4 = often. The total score ranges between 18 and 72. A higher score indicates a greater disturbance in the biological rhythm. The scale was translated from English to Arabic, back‐translated, and then approved by two Arabic native speakers who work in educational psychology.

Experts were shown a trial version of the Arabic version of the BRIAN in order to assess its clarity and appropriateness, as well as the relevance of the items and dimensions (sleep, activities, social rhythm, and eating patterns). The item was considered valid with over 80% agreement between the experts. In their evaluation, they recommended that four items should be rewritten. Those notes suggested by the experts were considered and implemented.

#### 2.2.2 Beck depression inventory (BDI‐II)

The current study used the Jordanian version of the BDI‐II developed by Al‐Da`asin.^25^ The scale consists of 21 questions for each question and a graded series of four alternatives (except two items 16 and 18 that consist of seven alternatives) representing the severity of depression arranged according to its severity from 0 to 3. Participants were asked to choose a phrase that best described their sentiments and condition throughout the previous 2 weeks. For example, with regard to their current levels of sadness, they could choose (0) I do not feel sad, (1) I feel sad, (2) I am sad all the time, and (3) I am too sad to be intolerable, with a total score ranging from 0 to 63. The score of each question is the number of the statement chosen by the participant. A high score indicates that you really are depressed. In the present study, BDI‐II had acceptable reliability (Cronbach’s α was 0.85).

#### 2.2.3 Sleep disorders inventory (SDI)

To measure the degree of sleep disorders, the sleep disorders inventory (SDI) developed by Al‐Jawarneh et al^7^ was used. The scale consisted of 29 items, divided into six disorders (insomnia, hypersomnia, sleep and wakefulness schedule, dreams during sleep, wandering during sleep, and speech during sleep). Each item has three responses listed from (1) never to (3) mostly. The overall score ranges between 29 and 87. A higher score indicates a high level of sleep disorders. In the present study, SDI had acceptable reliability (Cronbach’s α was 0.88).

### 2.3 Data analysis

The Statistical Package for Social Science (SPSS) version 26.0 was used to analyze the data. Descriptive statistics were used to describe the participants' demographic characteristics. To verify the psychometric properties of the scale, the classical theory of measurement (CTT) was used. The mean (M), standard deviation (SD), skewness (S), and kurtosis (K) values of the variables were calculated. CTT was used to verify the reliability of internal consistency through Cronbach’s α, and the Pearson Correlation Coefficient was used to calculate the correlation between the BRIAN, BDI‐II, and SDI.

The BRIAN factor structure was evaluated using exploratory factor analysis (EFA) with principal component analysis (PCA) and Promax rotation. The fit of the BRIAN was assessed using a confirmatory factor analysis (CFA) framework, which was analyzed in Amos for SPSS version 26.0. Robust maximum likelihood was used. A comparative fit index (CFI) ≥ 0.90, Tucker‐Lewis index (TLI) close to 0.95, Incremental Fit Index (IFI) ≥0.90, root mean square error of approximation (RMSEA) between 0.05 and 0.08 Lin et al,^26^ and standardized root mean square residual (SRMR) below 0.08 were thought to be a good fit.^27^, ^28^ To test the predictors of depression and sleep disorders, linear regression analysis was used.

## 3 RESULTS

Overall, 200 depressed people (mean score on BDI‐II 45.5 with range (39–61.8) and 203 healthy people (mean score on BDI‐II 22.4 with range (10–37) (for the control group) comprised the sample (women being 40.9% of it), with the mean age being 33 years ±10.2 and no statistically significant differences in any demographic characteristics between the two groups (Table 1).

**TABLE 1:** Demographic characteristics and descriptive statistics of the participants (*N* = 403)

	Depressive (*N* = 200)	Control group (*N* = 203)
	Mean ± SD or *n* (%)	
Age (years)	33.2 ± 10.5	32.6 ± 10.23
Gender		
Male	119 (59.5%)	119 (58.6%)
Female	81 (40.5%)	84 (41.4%)
Educational level		
Primary	26 (13%)	29 (14.3%)
Secondary	29 (14.5%)	38 (18.7%)
Bachelor’s	11 (5.5%)	15 (7.4%)
Master	52 (26%)	38 (18.7%)
Doctorate	82 (41%)	83 (40.9%)
Marital status		
Single	55 (27.5%)	67 (33%)
Married	123 (61.5%)	104 (51.2%)
Divorced	22 (11%)	32 (15.8%)
Employment status		
Yes	136 (68%)	121 (59.6%)
No	64 (32%)	82 (40.4%)

Descriptive statistics, such M, SD, S, and K were used to analyze data for all measurement tools at the overall score level (Table 2). All statistics met the criteria for a normal distribution.^29^, ^30^

**TABLE 2:** Comparison of the depressive and control groups on BRAIN dimensions, depression, and sleep disorders

Variables	Depressed mean (SD)	Control group mean (SD)	*t*	*P* value
BRIAN dimensions:				
1. Sleep	14.5 (5.5)	9.9 (6.2)	7.9	<0.0001
2. Eating Patterns	10.7 (3.8)	6.9 (6.2)	9.6	<0.0001
3. Social Rhythm	9.9 (3.5)	5.6 (3.3)	12.5	<0.0001
4. Activities	11.8 (3.7)	5.9 (2.3)	18.9	<0.0001
Total Score	46.9 (6.1)	28.4 (10.8)	21.2	<0.0001
BDI‐II	45.5 (5.8)	22.4 (9.1)	30.2	<0.0001
SDI	44.6 (7.3)	26.01 (10.5)	20.6	<0.0001

Abbreviations: BDI‐II, Beck Depression Inventory‐II; SDI, Sleep Disorder Inventory.

**FIGURE 1:**
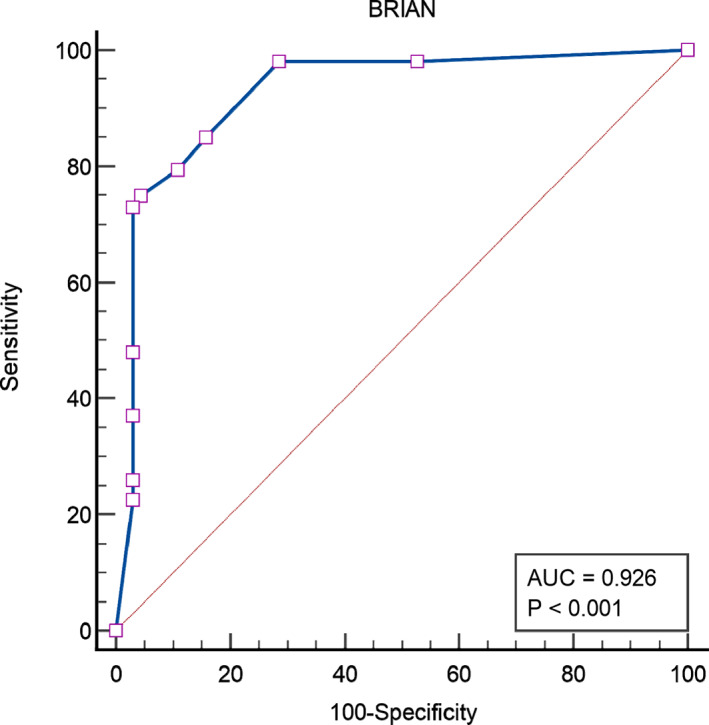
Receiver operating characteristic (ROC) curve

BRIAN was administered to all participants to extract its psychometric properties. It exhibited significant accuracy in distinguishing between depressed and non‐depressed people, as well as good specificity. For example, at cutoff 44, specificity was excellent, with a sensitivity of 75 (95.57). Table 3 shows the performance of BRIAN as a depression screening tool at different cutoff points. Figure 1 illustrates the results as a Receiver Operating Characteristic (ROC) Curve. The area under the curve (AUC) is 0.926.

**TABLE 3:** Accuracy of BRIAN as screener for depression disorder

Cutoff	Sensitivity	1‐specificity	Specificity	+LR	−LR	PPV	NPN
≥18	100.00	1.00	0.00	1.00		49.6	
>18	98	52.71	47.29	1.86	0.042	64.7	96
>32	98	28.57	71.43	3.43	0.028	77.2	97.3
>39	85	15.76	84.24	5.39	0.18	84.2	85.1
>43	79.5	10.84	89.16	7.34	0.23	87.8	81.5
>44	75	4.43	95.57	16.92	0.26	94.3	79.5
>45	73	2.96	97.04	24.7	0.28	96.1	78.5
>47	48	2.96	97.04	16.24	0.54	94.1	65.4
>53	0.00	0.00	100		1.00		50.4

Abbreviations: +LR, Positive Likelihood ratio; −LR, Negative Likelihood ratio; PPV, Positive Predicted Value; NPV, Negative Predicted Value.

The Cronbach’s value for the total score was 0.91, indicating that the internal consistency coefficients were excellent, sleep/social field = 0.91, activity = 0.94, and eating patterns = 0.92. The Pearson’s correlation coefficient between the BRIAN dimensions was 0.28 to 0.49 with statistical significance being reached at *p* < 0.01 (Table 4). The mean scores of the depressed group were higher than the control group on the overall score and BRIAN’s four subdomains. On all sub‐domains, the t test results demonstrated significant differences between the two groups Table 2. This result supported the discriminant validity of the BRIAN. There were no statistically significant differences between men and women on BRIAN’s overall score and sub‐dimensions (*t* = −0.10, *P* = 0.92).

**TABLE 4:** Correlation and descriptive statistics of the BRIAN dimensions, BDI‐II, and SDI (*N* = 403)

	M	SD	S	K	1	2	3	4	5	6	7
1. Sleep	12.18	6.31	0.17	−1.74	1						
2. Eating patterns	8.83	4.40	0.18	−1.69	0.30*	1					
3. Social rhythm	7.75	4.03	0.59	−1.24	0.28*	0.30*	1				
4. Activities	8.85	4.26	0.55	−1.19	0.30*	0.48*	0.40*	1			
5. Total score	37.60	12.78	−0.52	−1.20	0.66*	0.57*	0.69*	0.77*	1		
6. BDI‐II	33.91	13.87	−0.29	−1.01	0.52*	0.51*	0.65*	0.71*	0.88*	1	
7. SDI	35.25	13.00	−0.47	−1.09	0.57*	0.47*	0.59*	0.59*	0.83*	0.82*	1

* Statically significant at *p* < 0.01 (two‐tailed).

One of the main aims of the ongoing study was to verify the correlation between depression and sleep disturbances with the BRIAN’s overall score as well as with its sub‐measures. Table 4 indicates which variables are related to each other. The results revealed moderate to high positive correlations between depression and BRIAN and its four sub‐measures, ranging between 0.51 and 0.88 (*P* < 0.01). Medium to high positive correlations between SDI and BRIAN and its four sub‐measures ranged between 0.47 and 0.83 (*P* < 0.01). This result supported the concurrent validity of the BRIAN.

Standard regression analysis, considering the total score of BRIAN as a dependent variable and each of the depression and sleep disorders separately as an independent variable, revealed that depression accounted for 76.8% (*β* = 0.87) of the BRIAN’s overall score variance. Sleep disturbance accounted for 68.7% (*β* = 0.83). In summary, the current study showed that the total score of the BRIAN and its four sub‐dimensions correlated with depression and sleep disturbances. This result supported the construct validity of the BRIAN (see Table 4).

To reveal the factor structure of the BRIAN, an EFA was performed on a healthy group (*n* = 203) using the principal components method with Promax rotation. Three‐factor solutions were observed. The sleep/social factor was loaded with nine items, the activity factor was loaded with five items, and the eating factor was loaded with four items. This result is similar to the original scale, except for the items related to sleep (items 1–5), which also loaded on the second factor (activity), but with fewer loading values than on the sleep/social factor (see Table 5).

**TABLE 5:** EFA of the BRIAN

Factors	Sleep/social	Activity/sleep	Eating
BRIAN1	0.83	0.72	
BRIAN2	0.91	0.64	
BRIAN3	0.83	0.72	
BRIAN4	0.83	0.72	
BRIAN5	0.82	0.74	
BRIAN11	0.95		
BRIAN12	0.83		
BRIAN13	0.95		
BRIAN14	0.95		
BRIAN6		0.98	
BRIAN7		0.98	
BRIAN8		0.98	
BRIAN9		0.98	
BRIAN10		0.98	
BRIAN15			0.97
BRIAN16			0.83
BRIAN17			0.97
BRIAN18			0.97

A CFA was performed on the depressed group (*N* = 200). It showed an excellent match for the three expected theoretical factors: *χ* ^2^ = 2.82, *P* = 0.09, CFI = 0.99, TLI = 0.96, IFI = 0.99, RMSEA = 0.067 < 0.8, and SRMR = 0.022. The CFA findings backed up the BRIAN’s conceptual validity.

## 4 DISCUSSION

In this cross‐sectional study, we presented the development of a new version of the BRIAN, an Arabic version of BRIAN‐A, using the CTT. The results showed that the BRIAN‐A represents a consistent and valid tool for measuring biological rhythm. Additional important results were presented related to factor structure, discriminant validity, and concurrent validity on a sample taken from an Eastern environment, which differs from the Western environment in which most of the previous studies were conducted, and among a group of people who had not been clinically diagnosed with mental or psychological disorders. The Arabic version of BRIAN produced commendable results in terms of discriminant validity when screening depression cases. Because of the high discriminative validity, we can achieve an excellent performance when using BRIAN as a screening tool at the cutoff 44.

The screening tool is useful if it does not produce false positives and thus has a high negative predictive value. From this vantage point, the negative predictive value we discovered at the cutoff 44 is intriguing.

The internal consistency of the Arabic version of BRIAN, its overall score, and the scores of the sub‐dimensions were excellent and similar to those observed on the original version (Giglio et al^19^ and higher than the ones recorded on other translated versions,^31^, ^32^, ^33^ making it a reliable diagnostic tool for estimating biological rhythm disturbances. The main finding of the current study was that depressed people suffer from biological rhythm disturbances when subjected to a ban on movement due to the lockdown to limit the spread of the coronavirus greater than healthy subjects when exposed to the same conditions, as measured by the BRIAN scale.

The overall score of these depressed people was higher on the overall scale and the four sub‐scales, (social, sleep, activities, and eating). This is consistent with the results of previous studies.^10^, ^11^, ^19^, ^23^ Significant differences were also observed on the SDI scale between depressed patients and the control group. The items of the scale were consistent across age groups and gender, and the more severe the disturbance in rhythms, the higher the scores in the inventory of depression and for sleep disorders. The pattern of responses on the scale was unaffected by gender or age; this was slightly similar to the results of previous studies.^6^, ^10^, ^19^

The factor structure was verified by performing an EFA and CFA on the different samples. The EFA was performed on the control group consisting of healthy people who underwent a period of work interruption and stayed at home as a result of the epidemic of COVID‐19. We expect that they will face a disturbance in their biological rhythm as a result of these conditions. The most important result was the repetition of the three‐factor solution as in the original Giglio et al,^19^ Turkish Aydemir et al,^31^ and Japanese versions Kanda et al^32^ It appeared that the composition of the biological rhythm is similar across many countries and in various Eastern and Western cultures. Except for the sleep factor, which was loaded with two factors at the same time, on the social, which is called the sleep/social factor, also loaded on activity (activity/sleep), all the loading of items were less than their loading on the sleep/social factor (see Table 5). This may be due to the nature of the exceptional conditions that the sample members were exposed to; they were forced to sit at home, unable to carry out their normal activities, without social interaction due to the lockdown for the COVID‐19 pandemic. Thus, their biological rhythm was disturbed, so sleep was correlated with a lack of activity and social pattern at the same time.

The results of the CFA confirmed the validity of the BRIAN construct. It was similar to the theoretical construct as expected. The indicators of the good fit of the model were acceptable. The summary of the two results provided evidence of the possibility of using BRIAN in healthy people and patients. The intrinsic positive correlations between BRIAN‐A, depression (BDI‐II), and sleep disorders (SDI) gave evidence for its concurrent validity. The BRIAN sub‐scales also correlated as expected with depression and sleep disorders; this result was consistent with the biological rhythms theory.

Concerning the criterion validity, BRIAN appeared to be a reliable predictor of both depression and sleep disturbances, as revealed by the results of the regression analysis. Nonetheless, this study provided a significant advance to biological rhythm measurement by giving evidence that supports both the theoretical construction of the concept and its capability to predict two essential criteria of depression and sleep disturbance.

This study has some limitations. The limited sample size and the target population who underwent home quarantine did not allow the normative standardization of the Arabic version of BRIAN. Another limitation is that we only looked at biological rhythm disruptions that were reported in a self‐administered questionnaire that assessed an individual’s biological rhythm patterns over the home quarantine for 14–28 days. This data may be biased due to subjective differences in assessment and the depressed group’s exaggerated perceptions of circadian rhythm disturbances.

In light of the findings of the current study, which showed good psychometric properties of the Arabic version of BRIAN, we recommend that a new longitudinal study be conducted on a sample larger than the current sample and of different ages, in addition to the inclusion of kids under the age of 18 years in the sample. The results showed a good correlation between the severity of depression and sleep disorders, and we suggested that studies should be conducted to reveal the relationship between biological rhythm disturbances and other psychological disorders such as the quality of life, mood disorders and level of alertness. Although further research is needed to establish causation, these findings suggest that increased physical activity is an important and realistic step in the treatment of depression and psychological disorders.

## AUTHORS’ CONTRIBUTIONS

OSM and KAA were involved in Conceptualization, Data curation, Formal analysis, Investigation, Methodology, Project administration, Supervision, Validation, Visualization, Writing – Original draft, Methodology, Writing – Review & editing. Both authors contributed to and approved the final article.

## CONFLICT OF INTEREST

*The authors declare no conflict of interest*.

## ETHICAL APPROVAL

Procedures were followed in accordance with the ethical standards of the responsible committee on human experimentation and with the Helsinki Declaration.

## APPROVAL OF THE RESEARCH PROTOCOL BY INSTITUTIONAL REVIEW BROAD

The recommendations of Scientific Research Ethics Committee of Jordanian Universities have been followed after obtaining the approval of Al‐Balqa Applied University to conduct the research.

## INFORMED CONSENT

All participants provided their written informed consent.

## REGISTRY AND THE REGISTRATION NUMBER OF THE STUDY/TRIAL

Not available.

## ANIMAL STUDIES

Not available.

## Data Availability

The data that support the findings of this study are available on request from the corresponding author. The data are not publicly available due to privacy or ethical restrictions, because the authors have not obtained informed consent to disclose raw data.
